# Muscle PGC-1α modulates satellite cell number and proliferation by remodeling the stem cell niche

**DOI:** 10.1186/s13395-016-0111-9

**Published:** 2016-12-02

**Authors:** Ivana Dinulovic, Regula Furrer, Markus Beer, Arnaud Ferry, Bettina Cardel, Christoph Handschin

**Affiliations:** 1 Biozentrum, University of Basel, Klingelbergstrasse 50/70, 4056 Basel, Switzerland; 2 Thérapie des maladies du muscle strié INSERM U974 - CNRS UMR7215 - UPMC UM76 - Institut de Myologie and University Rene Descartes, 47 bld de l’Hôpital, G.H. Pitié-Salpétrière, 75013 Paris, France

**Keywords:** PGC-1α, Satellite cells, Satellite cell niche, Basal lamina, Fibronectin, Skeletal muscle

## Abstract

**Background:**

The myogenic capacity of satellite cells (SCs), adult muscle stem cells, is influenced by aging, exercise, and other factors. In skeletal muscle, the peroxisome proliferator-activated receptor γ coactivator 1α (PGC-1α) is a key regulator of oxidative metabolism and endurance training adaptation. However, a link between PGC-1α and SC behavior remains unexplored.

**Methods:**

We have now studied SC function in a PGC-1α fiber-specific gain-of-function animal model.

**Results:**

In surprising contrast to bona fide exercise, muscle-specific PGC-1α transgenic mice have lower SC numbers. Nevertheless, SCs from these mice have a higher propensity for activation and proliferation. Intriguingly, muscle PGC-1α triggers a remodeling of the SC niche by altering the extracellular matrix composition, including the levels of fibronectin, which affects the proliferative output of SCs.

**Conclusions:**

Taken together, PGC-1α indirectly affects SC plasticity in skeletal muscle and thereby might contribute to improved SC activation in exercise.

**Electronic supplementary material:**

The online version of this article (doi:10.1186/s13395-016-0111-9) contains supplementary material, which is available to authorized users.

## Background

Satellite cells (SC) are adult muscle stem cells located at the periphery of muscle fibers between the sarcolemma and basal lamina (BL) [1], in the vicinity of blood vessels and the neuromuscular junction [2]. SCs are accordingly exposed to various signals from within and outside of the fiber, which collectively comprise the specific environment termed the SC niche. Although metabolically inactive and quiescent in resting conditions, SCs quickly become activated in response to a stimulus such as injury or strenuous exercise [3]. These stem cells are indispensable for skeletal muscle regeneration [4], and despite being present in relatively small numbers (2–5% of total myonuclei), SCs have a vast proliferative and regenerative potential [3].

Proper activation and proliferation, as well as return to quiescence, are all essential to preserving SC number and function. In various pathological contexts, for example, in certain muscular dystrophies or aging, a depletion of SC numbers is linked to impaired regenerative capacity [5]. Importantly, reduced SC numbers and myogenic activity are often caused by alterations of the SC niche. For example, excess fibronectin (FN) in the BL in an uninjured state is correlated with a reduced ability of SCs to respond to injury [6]. Age-associated accumulation of extracellular matrix (ECM) components leads to the thickening of the BL [7], thereby preventing SCs from sensing changes in the environment and resulting in a reduced activation propensity. Inversely, treatment with fibronectin can restore satellite cell activation in old muscle [8]. Moreover, local transient FN secretion by SCs is an important step in the cascade of SC activation and subsequent proliferation [9], and such a transient increase in FN muscle expression is necessary for successful regeneration [8].

SC numbers vary by muscle fiber type, with higher counts present in oxidative compared to glycolytic muscle beds [10]. In line with this, endurance exercise increases SC numbers in mice and humans [11, 12]. The peroxisome proliferator-activated receptor γ coactivator 1α (PGC-1α) is a major driver of oxidative fiber-type specification, mitochondrial biogenesis, and high endurance capacity [13]. Furthermore, PGC-1α gene expression is induced by exercise and exhibits a preference for slow, oxidative fibers [14]. Finally, muscle-specific overexpression of PGC-1α protects against a variety of muscle-wasting conditions, including fiber atrophy or the pathologies in dystrophic mouse models [15]. Nevertheless, a potential link between PGC-1α, oxidative fibers, exercise, and SCs has not been studied yet. By using a mouse model which specifically overexpresses PGC-1α in adult muscle fibers, we attempted to delineate the aforementioned missing link and assess the importance of indirect effects of PGC-1α on SC phenotype.

Here, we show that muscle fiber PGC-1α modulates SC number as well as proliferation and that the latter, at least in part, could be regulated by the altered expression of ECM components, including FN protein levels, in the BL. Increased PGC-1α content in the SC niche therefore results in an accelerated SC response to injury and higher myogenic capacity.

## Methods

### Animals

Muscle-specific PGC-1α-transgenic animals that express transgenic PGC-1α under the control of the muscle creatine kinase (MCK) promoter have been described (mTGs) [14]. Male 8–16 week-old animals were used unless stated otherwise. All animal experimental procedures performed in this study were approved by the Cantonal and institutional authorities.

### Cardiotoxin injury

Mice were anesthetized using an O_2_/sevoflurane gas mixture (3% sevoflurane). The lower part of the hind limbs was shaved and the belly region of the tibialis anterior (TA) muscle injected with the control vehicle (30 μl of PBS) or cardiotoxin (30 μl containing 3 μg of CTX, C9759 Sigma) using insulin syringes (U-100, 300 μl, 29G × 1/2 in.). Mice were sacrificed and muscles collected at various time points. In the case of multiple CTX injuries — our model of chronic injury — CTX was injected three times (with a 3-week period of rest between each injection) and muscles were collected 3 weeks after the last injury. TAs were transversally cut into two pieces and frozen for histology or RNA isolation.

### Immunohistochemistry (IHC)

The TA, extensor digitorum longus (EDL), and soleus (SOL) muscles were collected immediately after sacrifice, covered by OCT (Tissue-Tek, Sakura), and frozen in isopentane precooled in liquid nitrogen. Frozen muscles were cut into 8 μm-thick sections using a cryostat (Leica CM1950) and kept at −20 °C until staining. The sections were dried at room temperature (RT), fixed in 4% paraformaldehyde (PFA), permeabilized in methanol, and antigen-retrieved in a citrate buffer using a microwave oven. After blocking (1 h at RT) and overnight (o/n) incubation at 4 °C with primary antibodies, the sections were washed with PBS, incubated with secondary antibodies for 1.5 h at RT, then washed again, and mounted with Vectashield including DAPI. Blocking was performed using 1:100 diluted mouse IgG Fab fragment (015-000-007, Jackson ImmunoResearch) in 3% bovine serum albumin (BSA) in PBS. The primary antibodies used are Pax7 (DSHB) and laminin (ab11575 Abcam). The secondary antibodies used are biotin goat anti-mouse IgG Fcγ1 (115-065-205 Jackson ImmunoResearch), streptavidin Alexa568 (S11226 Invitrogen), and goat anti-rabbit Alexa488 (A11008 Invitrogen).

### Fiber isolation, fiber culture, and immunocytochemistry

Muscle fibers were isolated from the EDL muscle of 12–16-week-old animals as described in detail elsewhere [16]. Briefly, isolated muscles were digested in 2 mg/ml collagenase A (Roche Diagnostics) solution (DMEM, 2 mM l-glutamine, 1% antibiotic cocktail) for 1.5 h and fibers further separated using trituration with glass Pasteur pipettes. Single fibers were fixed either immediately (T0) in 10% PFA or after a 3-day incubation (T3) in culture media (DMEM, 2 mM l-glutamine, 10% fetal bovine serum, 0.5% chicken embryo extract, 1% antibiotic cocktail). Fixed fibers were permeabilized in 0.5% Triton X-100 in PBS, blocked with 20% horse serum in PBS for 30 min at RT, and incubated with the primary antibodies against Pax7 (DSHB) and MyoD C-20 (sc-304) as well as DAPI o/n at 4 °C. After washing with PBS, fibers were incubated with the secondary antibodies goat anti-mouse IgG1 Alexa568 (A21124 Invitrogen) and goat anti-rabbit Alexa488 (A11008 Invitrogen) for 1.5 h at RT, washed with PBS, and mounted on glass slides with Vectashield.

### Myoblast isolation, culture, and immunocytochemistry

Myoblasts (MBs) were isolated from the EDL of 2–3 week-old male mice using a single fiber-based technique [17]. EDL fibers were separated using collagenase A (Roche Diagnostics) and incubated in plastic dishes coated with matrigel (BD Biosciences). After several days, proliferating myoblasts emerging from fibers were propagated in culture media (HyClone DMEM, 10% horse serum, 20% fetal bovine serum, 1% chicken embryo extract, 2 mM l-glutamine, 5 ng/ml basic fibroblast growth factor, 1% antibiotic cocktail) and used for assessing myoblast proliferation rates. For that purpose, equivalent numbers of cells were plated on plastic dishes in low concentration and fixed in 4% PFA after different incubation periods (e.g., 2 and 4 days post-plating). Cells were permeabilized in 0.5% Triton X-100 in PBS, blocked with 3% BSA in PBS, and incubated o/n at 4 °C with the primary antibody against desmin (ab15200) in 3% BSA in PBS. After washing with PBS, cells were incubated for 1 h at RT with the secondary antibody goat anti-rabbit IgG Cy3 (111-165-045 Jackson ImmunoResearch), washed with PBS, and mounted using Vectashield with DAPI.

### Image acquisition and quantification

For IHC, images covering the whole area of muscle transversal sections were captured using a Zeiss LSM700 microscope with the Zen 2010 software and a 25x objective with a 0.5 zoom. SC numbers (Pax7^+^/DAPI^+^) were expressed per area unit for all muscles. Section area was measured using the ImageJ software and fiber Feret minimum assessed using the Analysis or ImageJ software. In the fiber culture experiment, satellite cell and myoblast numbers were quantified on approximately 60 fibers/mouse for T0 or approximately 20 fibers/mouse for T3 and expressed per fiber. In the case of isolated MBs, single cells were quantified prior to fixation on 10 random fields on a Leica DMI4000B microscope using a 10x objective or alternatively after staining (desmin^+^/DAPI^+^) on 100 fields on a Zeiss LSM700 microscope using a 10x objective. MB quantification was done by hand or with the help of the Imaris software. All quantifications were performed in a blind manner.

### RNA extraction and semiquantitative PCR (qPCR)

RNA was isolated from muscles using lysing matrix tubes (MP Biomedicals) and TRI Reagent (Sigma) according to the manufacturer’s instructions, and concentration was measured with a Nanodrop 1000 (Thermo Scientific). One microgram of RNA was digested with DNase I (Invitrogen), after which complementary DNA (cDNA) synthesis was performed by reverse transcription with Superscript II (Invitrogen). qPCR measurements were done on a StepOne instrument with SYBR green-based detection and used for assessing relative gene expression levels. TATA-binding protein (TBP) served as a housekeeping gene, and relative expression was calculated with the ΔΔCt method. Additional file 1 lists all of the qPCR primers.

### Protein isolation and immunoblot analysis

Protein isolation and immunoblot analysis was performed as described previously [18]. Briefly, muscle tissue was homogenized in ice-cold RIPA buffer and protein concentration determined by Bradford Assay. After diluting and denaturating the samples in Laemmli Sample Buffer, 30 μg protein per sample was run on an 8% SDS-polyacrylamide gel. To improve the transfer of larger proteins to the nitrocellulose membrane, the ethanol concentration in the transfer buffer was reduced to 10% and SDS was added to a final concentration of 0.1%. Following the 90-min transfer at a constant voltage of 100 V, protein transfer was verified by Ponceau staining. The membrane was blocked in 5% BSA for 60 min and incubated overnight at 4 °C with primary antibodies. For protein detection, the following antibodies were used: fibronectin (FN, ab2413, Abcam; dilution 1/1000), α-tubulin (2125, Cell Signaling; dilution 1/1000), and polyclonal swine anti-rabbit immunoglobulins/HRP (P0399, Dako; dilution 1/3000 for FN and 1/10,000 for α-tubulin). After incubating the membranes in enhanced chemiluminescence reagent (Pierce 32106 or 34080), the western blots were visualized (Fusion Fix, Vilber Lourmat) and quantified (ImageJ software, National Institutes of Health). Samples were normalized to the expression of α-tubulin.

### Muscle contractility measurements in situ

In situ isometric muscle contractility as a method for assessing muscle regeneration has been previously described [15]. Briefly, pentobarbital solution (60 mg/kg) was intraperitoneally administered to mice in order to ensure deep anesthesia. The hind paws and knees were fixed to a platform and distal tendons of the TA muscles attached to an isometric transducer (Harvard Bioscience). 0.1-ms supramaximal square wave pulses were used for sciatic nerve stimulation, the contractions were measured at muscle length L0 (the length yielding maximum tension during tetanus), and the data were recorded and analyzed with the PowerLab software (4SP, AD Instruments). Maximal tetanic force (P0) and specific force (P0/weight) were determined by measuring muscle response to tetanic stimulation (6.25, 12.5, 25, 50, 100, and 143 Hz) and muscle mass. Fatigue resistance was expressed as the time needed for the P0 of the muscle to drop to half its initial value and was measured with one continuous contraction (50 Hz for 45 s). Overdose of pentobarbital solution was used to sacrifice the mice at the end of the experiment.

### Statistical analysis

Data are expressed as average (AV) ± SEM. Gene expression values from CTX-injected muscles were normalized to the PBS values by means of unpaired assessment unless stated otherwise. Statistical analysis was based on Student’s *t* test and used for comparison of two genotypes under the same conditions. *p* ≤ 0.05 was interpreted as significant.

## Results

### PGC-1α overexpression in muscle reduces SC numbers

**Fig 1:**
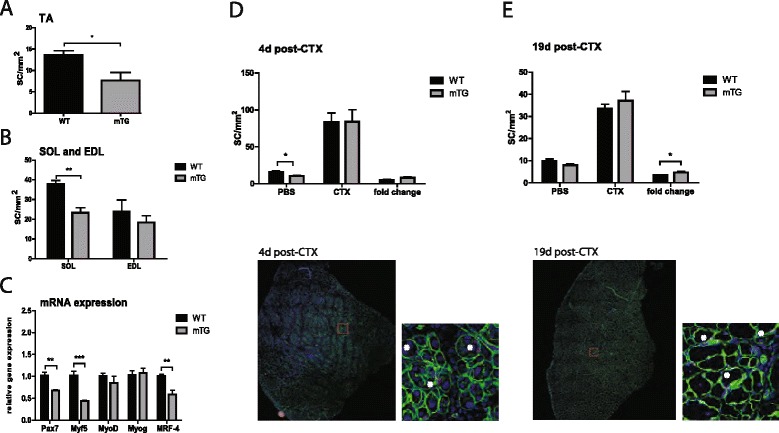
SC numbers, MRF expression, and SC proliferation after cardiotoxin injection. **a** SC numbers per area in TA (*n* = 4 per group), **b** SOL and EDL muscles (*n* = 4 per group) of mTGs and WTs. **c** Relative mRNA levels of Pax7 and MRFs in TA (*n* = 6 per group) of mTGs compared to controls (WT); mRNA levels in mTG were normalized to littermate control levels. **d** SC numbers per area 4 days post-CTX (*n* = 7–8 per group) and **e** 19 days post-CTX (*n* = 8–10 per group) in mTGs together with representative IHC images (laminin: *green*, DAPI/nuclei: *blue*; Pax7: *red*). *Red rectangles* denote enlarged areas of sections, and SCs are marked with *white asterisks*. Brightness/contrast was adjusted using ImageJ. Values are plotted as AV ± SEM; *p ≤ 0.05, **p ≤ 0.01, ***p ≤ 0.001

mTGs have muscles with a higher percentage of oxidative at the expense of glycolytic fibers [14]. The transgenic muscles are also rich in blood vessels and have greater endurance compared to wild-type (WT) muscles. Inversely, muscle-specific knockout mice exhibit the opposite phenotype [19]. In parallel to PGC-1α expression, SC numbers are higher in oxidative muscle beds [10] and can further be boosted by exercise [11, 12]. Thus, to directly test the effect of muscle PGC-1α on SCs, we assessed SC numbers in mTGs, a gain-of-function model for PGC-1α for which overexpression is restricted to the mature muscle fiber. For that purpose, the SCs (Pax7^+^/DAPI^+^ cells) on cross sections of TAs were quantified, and unexpectedly, lower SC numbers in mTGs were revealed (Fig 1a). A similar reduction in SC numbers was also observed in the soleus of mTGs compared to WT mice (Fig 1b), contrary to the specific enrichment of SCs in oxidative muscle beds that occurs in WT animals.

Next, the basal gene expression of Pax7 and myogenic regulatory factors (MRFs) in mTG TA muscles was determined (Fig 1c). Pax7 is a transcription factor that is specifically expressed in quiescent and activated SCs [20]. Upon activation, SCs increase in size, enter the cell cycle, and start expressing the MRFs Myf5 and MyoD, thereby becoming myoblasts. Some of the proliferating Pax7^+^/MyoD^+^ cells will continue on the differentiation path with an ensuing reduction in Pax7 and boost in myogenin gene expression. They will subsequently exit the cell cycle and form mononucleated postmitotic myocytes that will then, with increasing expression of MRF-4, start fusing in order to form myotubes. In mTGs, a decrease in the expression of the MRFs Myf5 and MRF-4 as well as Pax7 was detected (Fig 1c). Thus, the modulation of basal SC numbers was reflected in the regulation of MRF gene expression, in particular that of Pax7.

### PGC-1α levels affect SC proliferation after CTX injury

SCs are muscle stem cells that are activated upon injury, at which point they enter the cell cycle and proliferate in order to regenerate damaged muscle [3]. A decrease in SC numbers is a hallmark of chronic diseases such as Duchenne muscular dystrophy (DMD) but is also seen during aging [5, 21]. We therefore investigated whether the lower numbers of SCs in mTGs also affect the ability of muscle to cope with an acute injury. SCs in TA cross sections were quantified 4 and 19 days after CTX-induced muscle damage (Fig 1d, e). We observed a trend towards higher proliferative output in mTGs at 4 days post-CTX (Fig 1d). At the 19-day time point, a significant increase in the relative SC number in mTGs was recorded (Fig 1e). Thus, basal SC number did not correlate with SC proliferation upon muscle injury.

To study the time course of myogenesis in regeneration, we next measured Pax7 and MRF gene expression profiles in TA muscle during recovery after injury caused by CTX (Additional file 2). Similar to the altered SC activation seen in histological preparations, we observed exacerbated dynamics of the myogenic gene expression changes in injured compared to PBS-injected mTGs vis-à-vis the changes in injured compared to PBS-injected WT animals. Specifically, a higher increase in Pax7 and MyoD (Additional file 2A and 2B) in the early days of regeneration followed by their decline and a subsequent rise in expression levels of myogenin and MRF-4 (Additional file 2C and 2D) in the later days were detected in the mTGs vis-à-vis the more attenuated changes in the WTs. Notably, MRF gene expression changes in the two genotypes converged at later time points, e.g., at 19 days after CTX injection (Additional file 2). Accordingly, in situ contractility experiments did not reveal any significant differences between mTGs and WT mice in regard to absolute force (Additional file 3A), specific force (Additional file 3B), or fatigue resistance (Additional file 3C) at a late stage of muscle regeneration. This was also in line with the indistinguishable recovery of fiber sizes at 19 days after CTX injection (Additional file 3D and 3E) in the two genotypes. Thus, modulation of muscle PGC-1α seems to predominantly affect early stages of SC activation.

Given the observed increase in SC proliferative output in vivo upon injury in mTGs (Fig 1d, e), we wondered if this might perturb the processes of SC renewal and consequently lead to SC depletion, which would affect future rounds of regeneration in these mice. To address this question, we performed triple CTX injuries as a chronic injury model and assessed SC numbers as well as fiber size distribution 3 weeks after the last injection (Additional file 4). Although a certain reduction in SC proliferative output was found in mTGs (Additional file 4A), SCs were not lost even in this model of profound repetitive injury. Moreover, regeneration progressed without obstacles and even proceeded faster in mTGs as evidenced by larger fibers (Additional file 4B and 4C).

### PGC-1α modulates the SC niche

**Fig 2:**
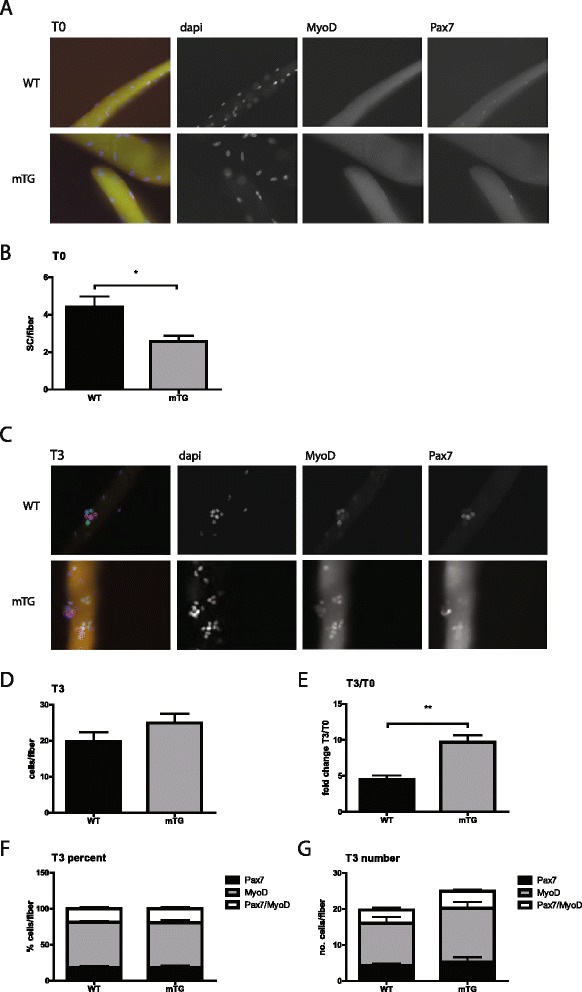
SC numbers and proliferation on fibers in culture. **a** Representative IHC images (DAPI/nuclei: *blue*; MyoD: *green*; Pax7: *red*) of SCs on freshly isolated fibers (T0) and **b** SC quantification per fiber in mTGs and WT mice. Approximately 60 fibers per mouse and 5 mice per group were used. **c** Representative IHC images (DAPI/nuclei: *blue*; MyoD: *green*; Pax7: *red*) of SC progeny on fibers kept for 3 days in culture media (T3). Quantification results: **d** Total proliferative output of SCs. **e** Normalized proliferative output (T3/T0). **f** Quantification of Pax7^+^/MyoD^−^, Pax7^+^/MyoD^+^, and Pax7^−^/MyoD^+^ cells per fiber expressed as percentages and **g** numbers of total proliferative output. Approximately 20 fibers per mouse and 5 mice per group were used. Brightness/contrast was adjusted using ImageJ. Values are plotted as AV ± SEM; *p ≤ 0.05, **p ≤ 0.01

Skeletal muscle regeneration is a very complex process that involves the participation of muscle fibers, SCs, and various other cell types [22]. To study the effects of muscle PGC-1α on the early events in SC activation, we used an ex vivo approach based on isolation and culture of single muscle fibers together with SCs in which SC numbers on freshly isolated fibers (T0) and the SC proliferative output after 3 days in culture (T3) were quantified (Fig 2). First, this ex vivo experiment revealed lower numbers of SCs (Pax7^+^/DAPI^+^) per fiber originating from mTG animals (Fig 2a, b), as observed in the study in vivo (Fig 1a). Next, the quantification of quiescent SCs (Pax7^+^/MyoD^−^/DAPI^+^), committed progenitors (Pax7^+^/MyoD^+^/DAPI^+^), and myoblasts (Pax7^−^/MyoD^+^/DAPI^+^) per fiber at T3 revealed a higher relative proliferative output (T3/T0) in mTGs (Fig 2c–g), again in line with the in vivo observations (Fig 1d, e).

**Fig 3:**
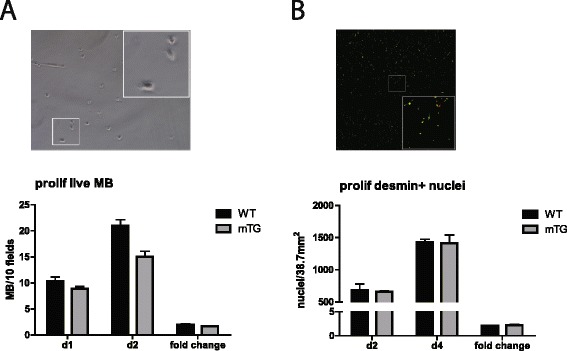
Myoblast proliferation outside of the SC niche. **a** Assessment of live mTG and WT myoblasts (MBs) 1 and 2 days after plating equal numbers and fold change in MB numbers between these two days. Cells were quantified on 10 fields per dish, 2 dishes per mouse, and 3 mice per genotype. Representative bright-field image with the *white rectangle* indicating enlarged area. **b** Desmin-positive cell quantification 2 and 4 days after plating equal number of MBs and fold change between these two days. Cells were quantified on an area of 38.7 mm^2^ per dish, 2 dishes per mouse, and 3 mice per genotype. Representative IHC image (DAPI/nuclei: *red*; desmin: *green*) with the *white rectangle* indicating enlarged area. Values are plotted as AV ± SEM

Subsequently, we tested the proliferative output of SCs outside of their niche. Primary myoblasts were isolated and their proliferative rate determined (Fig 3). The purity of the primary population was verified by desmin staining. Myoblast proliferation was assessed by comparing cell numbers between two incubation periods in order to minimize the influence of variations in the number of cells plated. Quantification was performed on live cells (Fig 3a), as well as on fixed and stained myoblasts (Fig 3b). In either case, we did not observe any differences in the proliferation rates between mTG and WT myoblasts. Thus, since the modulation of SC activation is lost in this experiment and not in the ex vivo fiber culture, the results together indicate a predominant role of the fiber-associated SC niche in mediating the observed effects of PGC-1α on SC proliferation rates.

### The stem cell niche influences SC proliferation in the PGC-1α mouse model

**Fig 4:**
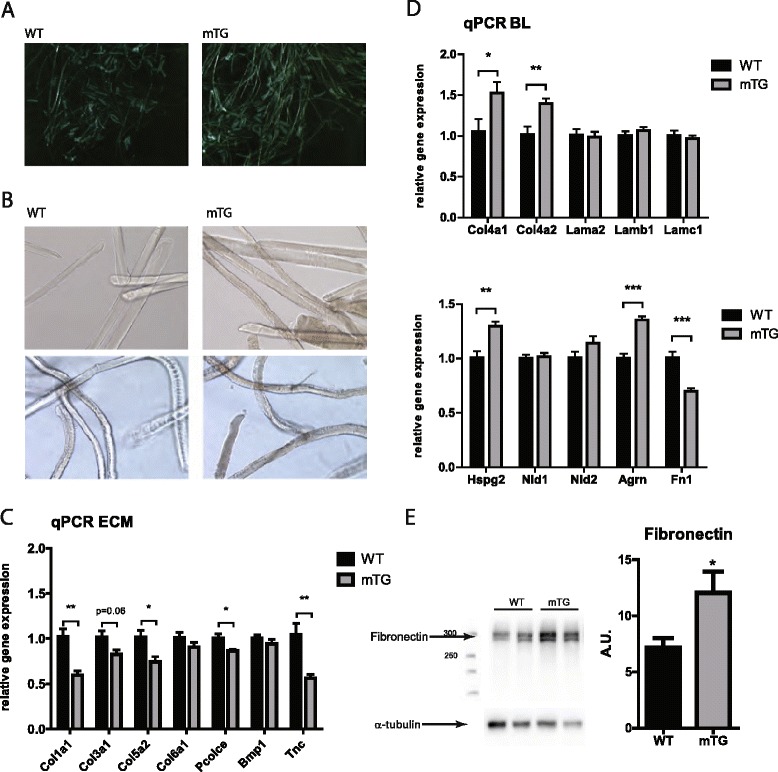
Extracellular matrix gene expression. **a** Representative images of freshly isolated fibers from mTGs and WT mice. **b** Images of WT and mTG fibers after 3 days in culture media, prior to fixation. WT fibers look translucent and silky while mTG fibers appear matte and with cracks on the surface. **c** Relative mRNA levels of ECM components in mTGs and WT mice (*n* = 6 per group). **d** Relative mRNA levels of basal lamina (BL) components in mTGs and WT mice (*n* = 6 per group). **e** Western blot analysis and quantification of fibronectin normalized to α-tubulin levels. Values are plotted as AV ± SEM; *p ≤ 0.05, **p ≤ 0.01, ***p ≤ 0.001

Intriguingly, we observed that the translucence of isolated fibers is vastly different between the PGC-1α gain-of-function and WT mice (Fig 4a, b). This prompted us to assess the composition of the ECM, which surrounds muscle fibers, by measuring the gene expression of collagens and glycoproteins (Fig 4c), in particular of those that comprise the closest layer to the SCs, also called the BL (Fig 4d). We noticed that several collagens are downregulated in mTGs (Fig 4c). In addition, reduced expression in two components of the BL, fibronectin (FN), and tenascin C (TNC) was found in mTGs (Fig 4c, d). To verify whether the reduction in messenger RNA (mRNA) expression of FN observed in mTGs corresponds to the protein levels, FN protein concentration was determined by western blot (Fig 4e). Surprisingly, in contrast to the reduction observed in gene expression, protein levels of FN were significantly elevated in mTG animals compared to WTs.

## Discussion

SC number, activation, and myogenic potential are modulated in different physiological and pathophysiological contexts. For example, the higher number of SCs in oxidative compared to glycolytic muscle beds [10] correlates with the boost in SC numbers by endurance exercise [11, 12]. We now demonstrate that the coactivator PGC-1α, a central regulatory nexus in training adaptation, modulates SC activation in animals in vivo and in isolated fiber/SC preparations ex vivo. Intriguingly, this was achieved in a model that limits PGC-1α overexpression to adult muscle fibers and therefore does not affect SCs directly. Accordingly, our data indicate that a PGC-1α-dependent remodeling of the SC niche affects SC activation upon injury.

As a potential explanation, our observations point towards PGC-1α-mediated adaptations in the composition of the ECM and the BL. Besides a change in collagen gene expression, we detected significantly reduced transcript levels of FN (~30% reduction) and TNC (~50% reduction) in mTG muscles. Surprisingly, despite this PGC-1α-dependent repression of FN gene expression, the protein levels of FN were markedly elevated in the mTG muscles. The cause of this disconnect between gene expression and protein levels is unclear. The imperative of FN elevation for SC proliferation and muscle regeneration, as well as its transient increase in expression upon injury, has been recently reported [8, 9]. Inversely, the complete loss of FN in the basal state leads to SC loss and reduced proliferation [8]. Ectopic application of FN to old muscles to counteract age-dependent attenuation of FN confers beneficial effects on satellite cell function [8]. In fact, some of the effects that are observed in this model are recapitulated by the mTGs, in particular the higher satellite cell activation potential. Elevation of FN within a physiologically beneficial window in the mTGs is implied by the absence of the hallmarks of pathological levels of FN, e.g., the fibrosis and BL thickening upon chronic elevation of FN in the uninjured state [7] or the elevated resting SC numbers and reduced SC proliferative potential on fibers ex vivo caused by the increased basal expression of FN in like-acetylglucosaminyltransferase myodystrophy (LARGE^myd^) mice [6].

Besides ECM remodeling, other factors could obviously also contribute to the observed phenotype. For example, as a member of the transforming growth factor β (TGFβ) family, myostatin (Mstn) is an important regulator of fibrosis [23] and muscle mass [24]. In addition, several studies have reported that Mstn reduces SC and myoblast proliferation [25–27]. Mstn seems to be expressed by quiescent SCs [26], but more importantly, exogenous Mstn can block SC activation and proliferation in vitro [27]. Intriguingly, Mstn is considerably downregulated in mTGs even in the basal state, prior to injury (Additional file 5). Thus, Mstn could mediate some of the effects of muscle PGC-1α on SC activation and thus provide an explanation for the regulation of Mstn by PGC-1α in the absence of changes in muscle mass in the mTGs [28]. However, the functional contribution of Mstn and potentially other secreted factors in this context will have to be studied in more detail.

Very surprisingly, the fiber-type shift mediated by muscle PGC-1α was not linked to an increase in SC numbers, as would be expected from more oxidative or endurance-trained muscle beds. In fact, transgenic overexpression of PGC-1α consistently resulted in lower SC counts, both in muscle in vivo and in fiber/SC preparations ex vivo. This divergence is one of the very few parameters for which the PGC-1α-overexpressing and bona fide oxidative fibers are clearly different. In regard to most other metabolic and contractile properties, skeletal muscles of mTG animals closely mimic the phenotype of oxidative muscles [29]. Thus, the mechanistic underpinnings that control the diametrically opposite outcome between PGC-1α-overexpressing fibers with low vis-à-vis oxidative and endurance-trained muscle with high SC count is unclear. Importantly, this reduction in SC numbers was however offset by the higher proliferative potential of SCs in the mTG animals. At least this aspect could represent the contribution of muscle PGC-1α to SC biology in exercise.

## Conclusions

In summary, we now for the first time provide evidence for a PGC-1α-dependent stem cell niche remodeling that alters SC activation and proliferation. We identified fibronectin as a potential niche factor influencing the SC response to injury, whose gene expression and protein levels are inversely modulated by PGC-1α. Increasing SC proliferation would be of great therapeutic benefit in various muscle-wasting pathologies and aging, where reduced SC myogenic capacity impairs effective muscle regeneration. More importantly, our results indicate that this outcome could be achieved without directly manipulating SCs. This could be especially advantageous in stem cell-based transplantation to optimize therapeutic solutions.

## Additional files

Additional file 1: List of qPCR primers.
Additional file 2: Kinetics of Pax7 and MRF gene expression after cardiotoxin injury. Relative mRNA expression of A) Pax7, B) MyoD, C) Myog, and D) MRF-4 2 days (*n* = 4–5 per group), 4 days (*n* = 7–8 per group), 7 days (*n* = 5 per group), 10 days (*n* = 8 per group), and 19 days (*n* = 8–10 per group) post-CTX injection in mTGs and WT mice. Expression levels from CTX samples were normalized to PBS sample levels of corresponding genotype. Values are plotted as AV ± SEM; **p* ≤ 0.05, ***p* ≤ 0.01, ****p* ≤ 0.001.
Additional file 3: In situ TA contractility measurements 16 days and fiber size distribution 19 days after cardiotoxin injection. A) Absolute maximal force (P0), B) specific force (P0/weight), and C) fatigue resistance in mTGs and WT mice 16 days after CTX; *n* = 7–8 per group. Feret minimum measurements 19 days after injections in D) PBS- and E) CTX-injected TAs; *n* = 8–10 per group; Values are plotted as AV ± SEM; * *p* ≤ 0.05.
Additional file 4: Regeneration after multiple cardiotoxin injections. A) SC numbers 3 weeks after the last injection in WT and mTG TAs and fold change normalized to triple PBS-injected samples. Feret minimum measurements after multiple injections in B) PBS- and C) CTX-injected TAs; *n* = 7–9 per group; Values are plotted as AV ± SEM; **p* ≤ 0.05, ***p* ≤ 0.01.
Additional file 5: Mstn expression in WT and mTG muscles. Relative Mstn mRNA levels in the basal state in TAs of WT mice and mTGs; *n* = 5–6 per group. Values are plotted as AV ± SEM; ****p* ≤ 0.001.

## Abbreviations

BL: Basal lamina
CTX: Cardiotoxin
FN: Fibronectin
mTGs: Muscle-specific PGC-1α transgenic mice
PGC-1α: Peroxisome proliferator-activated receptor γ coactivator 1α
SCs: Satellite cells
TA: Tibialis anterior
