# Supplementary material for: Muscle PGC-1α modulates satellite cell number and proliferation by remodeling the stem cell niche
Source: Skelet Muscle. 2016 Dec 2;6:39. doi: 10.1186/s13395-016-0111-9 (PMC5134094; doi:10.1186/s13395-016-0111-9)
Supplement: Additional file 1: — List of qPCR primers. [file 13395_2016_111_MOESM1_ESM.doc]

# Additional File

**Additional File 1. List of qPCR primers**

| **Target** | **Fwd primer (5' - 3')** | **Rev primer (5' - 3')** |
| --- | --- | --- |
| Agrn | GCG GTA CTT GAA AGG CAA AGA | CTC CAA AGC CAC CAA TTA CCA |
| Bmp1 | ACC CTC CAA GAC AGC ACT G | GGC TAC GGT ACA GGT CCA T |
| Col1a1 | GCT CCT CTT AGG GGC CAC T | CCA CGT CTC ACC ATT GGG G |
| Col3a1 | CTG TAA CAT GGA AAC TGG GGA AA | CCA TAG CTG AAC TGA AAA CCA CC |
| Col4a1 | TCC GGG AGA GAT TGG TTT CC | CTG GCC TAT AAG CCC TGG T |
| Col4a2 | AGT GCT ACC CGG AGA AAG GAG | CCC TGT AGT CCT GGG AAT CC |
| Col5a2 | TTG GAA ACC TTC TCC ATG TCA GA | TCC CCA GTG GGT GTT ATA GGA |
| Col6a1 | CTG CTG CTA CAA GCC TGC T | CCC CAT AAG GTT TCA GCC TCA |
| Fn1 | ATG TGG ACC CCT CCT GAT AGT | GCC CAG TGA TTT CAG CAA AGG |
| Hspg2 | TTC CAG ATG GTC TAT TTC CGG G | CTT GGC ACT TGC ATC CTC C |
| Lama2 | TCC CAA GCG CAT CAA CAG AG | CAG TAC ATC TCG GGT CCT TTT TC |
| Lamb1 | AGA CTT TGG GGG TTC ATG TCA | ATC GTC CCG TCT CCT TGT CA |
| Lamc1 | CTG TGA GAA CAC GTA CTC AAA GG | AGG GTT GAA AAG GCC ACG TT |
| MRF-4 | CGC GAA AGG AGG AGA CTA AAG A | CCA CAG TCC GAC GCT TCA G |
| Mstn | GCT GGC CCA GTG GAT CTA AA | CAG CCC CTC TTT TTC CAC ATT |
| Myf5 | CAT GTG GGC CTG CAA AGC | TGC GCC GAT CCA TGG TA |
| MyoD1 | GCC GGT GTG CAT TCC AA | CAC TCC GGA ACC CCA ACA G |
| Myog | GCA GCG CCA TCC AGT ACA TT | ATC GCG CTC CTC CTG GTT |
| Nid1 | CCC AGC TTC GGC TCA GTA G | AAC GGG GAT AAG TCT TCT CGA T |
| Nid2 | CTC TTT CCT TAC GGG GAG TCG | GGC ATC GTA GAA ACG CAG G |
| Pax7 | AAA AAA CCC TTT CCC TTC CTA CA | AGC ATG GGT AGA TGG CAC ACT |
| Pcolce | GCC AGA CCC CCA ACT ACA C | CCG TAA TTG TCC AGA TGC ACT T |
| TBP | TGC TGT TGG TGA TTG TTG GT | CTG GCT TGT GTG GGA AAG AT |
| Tnc | TTT GCC CTC ACT CCC GAA G | AGG GTC ATG TTT AGC CCA CTC |
