# Supplementary figures and images for: Muscle PGC-1α modulates satellite cell number and proliferation by remodeling the stem cell niche
Source: Skelet Muscle. 2016 Dec 2;6:39. doi: 10.1186/s13395-016-0111-9 (PMC5134094; doi:10.1186/s13395-016-0111-9)

A

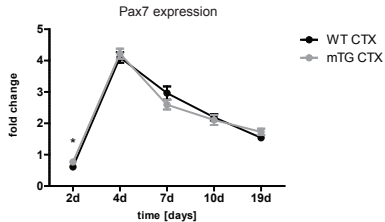

B

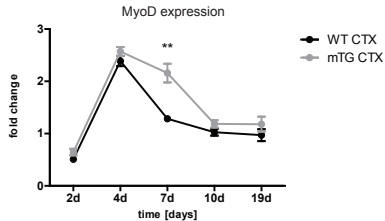

C

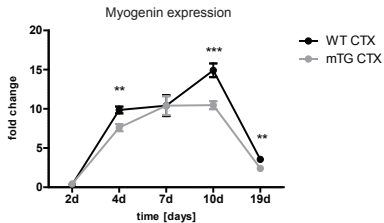

D

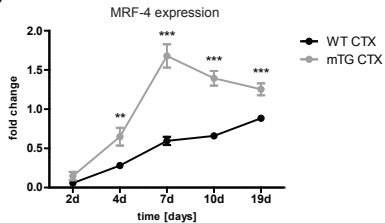

Supplement: Additional file 2: — Kinetics of Pax7 and MRF gene expression after cardiotoxin injury. Relative mRNA expression of A) Pax7, B) MyoD, C) Myog, and D) MRF-4 2 days (n = 4–5 per group), 4 days (n = 7–8 per group), 7 days (n = 5 per group), 10 days (n = 8 per group), and 19 days (n = 8–10 per group) post-CTX injection in mTGs and WT mice. Expression levels from CTX samples were normalized to PBS sample levels of corresponding genotype. Values are plotted as AV ± SEM; *p ≤ 0.05, **p ≤ 0.01, ***p ≤ 0.001. [file 13395_2016_111_MOESM2_ESM.pdf]

A

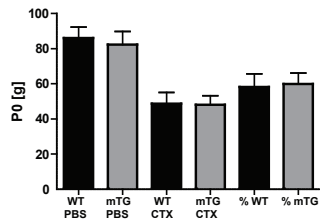

B

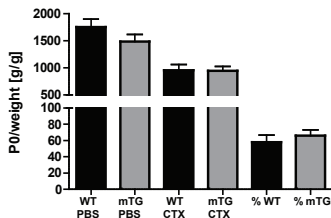

C

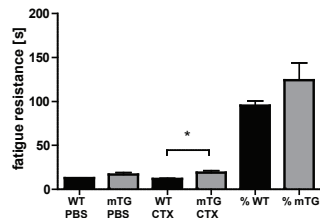

D

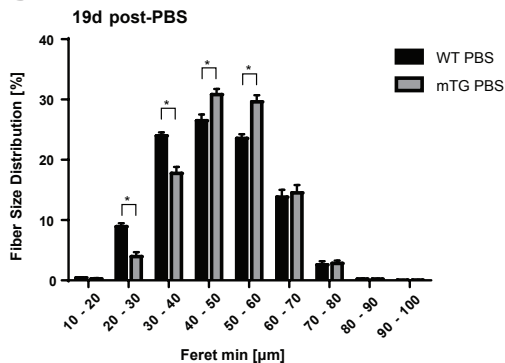

E

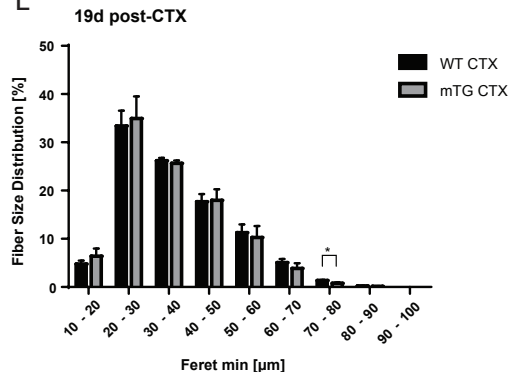

Supplement: Additional file 3: — In situ TA contractility measurements 16 days and fiber size distribution 19 days after cardiotoxin injection. A) Absolute maximal force (P0), B) specific force (P0/weight), and C) fatigue resistance in mTGs and WT mice 16 days after CTX; n = 7–8 per group. Feret minimum measurements 19 days after injections in D) PBS- and E) CTX-injected TAs; n = 8–10 per group; Values are plotted as AV ± SEM; * p ≤ 0.05. [file 13395_2016_111_MOESM3_ESM.pdf]

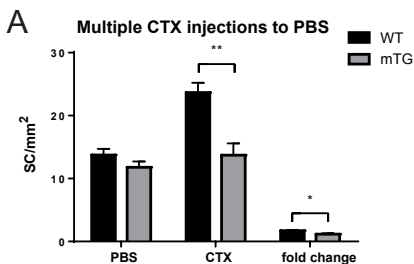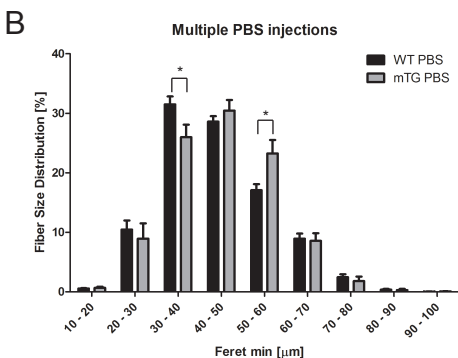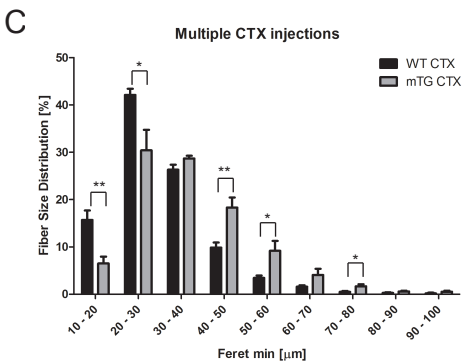

Supplement: Additional file 4: — Regeneration after multiple cardiotoxin injections. A) SC numbers 3 weeks after the last injection in WT and mTG TAs and fold change normalized to triple PBS-injected samples. Feret minimum measurements after multiple injections in B) PBS- and C) CTX-injected TAs; n = 7–9 per group; Values are plotted as AV ± SEM; *p ≤ 0.05, **p ≤ 0.01. [file 13395_2016_111_MOESM4_ESM.pdf]

## Mstn expression

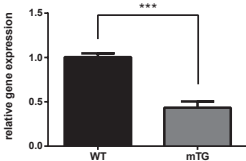

Supplement: Additional file 5: — Mstn expression in WT and mTG muscles. Relative Mstn mRNA levels in the basal state in TAs of WT mice and mTGs; n = 5–6 per group. Values are plotted as AV ± SEM; ***p ≤ 0.001. [file 13395_2016_111_MOESM5_ESM.pdf]
